# Combination of Mycobacterium tuberculosis RS Ratio and CFU Improves the Ability of Murine Efficacy Experiments to Distinguish between Drug Treatments

**DOI:** 10.1128/aac.02310-21

**Published:** 2022-03-21

**Authors:** Christian Dide-Agossou, Allison A. Bauman, Michelle E. Ramey, Karen Rossmassler, Reem Al Mubarak, Samantha Pauly, Martin I. Voskuil, Maria Garcia-Cremades, Rada M. Savic, Payam Nahid, Camille M. Moore, Rokeya Tasneen, Eric L. Nuermberger, Gregory T. Robertson, Nicholas D. Walter

**Affiliations:** a Department of Epidemiology, Colorado School of Public Health, Aurora, Colorado, USA; b Mycobacteria Research Laboratories, Department of Microbiology, Immunology, and Pathology, Colorado State University, Fort Collins, Colorado, USA; c Rocky Mountain Regional VA Medical Center, Aurora, Colorado, USA; d Division of Pulmonary Sciences and Critical Care Medicine, University of Colorado Anschutz Medical Campus, Aurora, Colorado, USA; e Department of Immunology and Microbiology, University of Colorado Anschutz Medical Campus, Aurora, Colorado, USA; f Consortium for Applied Microbial Metrics, Aurora, Colorado, USA; g Department of Bioengineering and Therapeutic Sciences, University of California San Francisco, San Francisco, California, USA; h Departamento de Farmacia Galénica y Tecnología Alimentaria, Facultad de Farmacia, Universidad Complutense de Madrid, Madrid, Spain; i Division of Pulmonary and Critical Care Medicine, University of California San Francisco, San Francisco, California, USA; j Division of HIV, Infectious Diseases and Global Medicine, University of California San Francisco, San Francisco, California, USA; k UCSF Center for Tuberculosis, San Francisco, California, USA; l Division of Biostatistics and Bioinformatics, National Jewish Health, Denver, Colorado, USA; m Center for Tuberculosis Research, Johns Hopkins University, Baltimore, Maryland, USA

**Keywords:** 16S rRNA burden, antimicrobial therapies, BALB/c relapse models, CFU, drug efficacy, murine drug experiments, pharmacodynamic marker, RS ratio, rRNA

## Abstract

Murine tuberculosis drug efficacy studies have historically monitored bacterial burden based on CFU of Mycobacterium tuberculosis in lung homogenate. In an alternative approach, a recently described molecular pharmacodynamic marker called the RS ratio quantifies drug effect on a fundamental cellular process, ongoing rRNA synthesis. Here, we evaluated the ability of different pharmacodynamic markers to distinguish between treatments in three BALB/c mouse experiments at two institutions. We confirmed that different pharmacodynamic markers measure distinct biological responses. We found that a combination of pharmacodynamic markers distinguishes between treatments better than any single marker. The combination of the RS ratio with CFU showed the greatest ability to recapitulate the rank order of regimen treatment-shortening activity, providing proof of concept that simultaneous assessment of pharmacodynamic markers measuring different properties will enhance insight gained from animal models and accelerate development of new combination regimens. These results suggest potential for a new era in which antimicrobial therapies are evaluated not only on culture-based measures of bacterial burden but also on molecular assays that indicate how drugs impact the physiological state of the pathogen.

## INTRODUCTION

There is an urgent need for shorter treatment regimens for both drug-susceptible and drug-resistant tuberculosis (TB). Murine models have historically been the backbone of preclinical evaluation of TB drugs and treatment regimens (1–3). Pharmacodynamic (PD) monitoring in murine drug experiments conventionally measures colony-forming units (CFU) in lung homogenate. Measurement of 16S rRNA burden has been proposed as an alternative measure of Mycobacterium tuberculosis burden (4–6). Importantly, neither change in CFU nor rRNA burden during the period that mice are administered treatment accurately indicates the treatment-shortening activity of drugs or regimens (1, 6, 7). Therefore, experiments evaluating the efficacy of multidrug regimens are commonly based on the proportion of mice with microbiologic relapse 12 weeks or more after treatment cessation (8, 9). Because determination of the relapse proportion requires large, resource-intensive mouse experiments sometimes lasting 9 to 10 months, the current standard experimental design is a critical bottleneck in TB regimen evaluation. To accelerate regimen evaluation, there is a need for a PD marker or combination of PD markers that indicate the treatment-shortening activity in shorter, less-resource-intensive murine experiments without the need for determination of relapse.

We recently proposed a novel molecular PD marker called the RS ratio (10). The RS ratio measures ongoing rRNA synthesis in M. tuberculosis by quantifying the abundance of M. tuberculosis precursor rRNA (pre-rRNA) relative to stable 23S rRNA. Unlike CFU, 16S rRNA burden, and other existing PD markers that enumerate the abundance of M. tuberculosis, the RS ratio measures the degree to which drugs and regimens interrupt rRNA synthesis. In the absence of drug treatment, RS ratio was validated as a surrogate for bacterial replication rate (10). In the presence of drug treatment, RS ratio differentiates individual drug or drug regimen effect *in vitro* and *in vivo* and, as such, may represent an important new PD marker (10).

In the current work, we used results from three BALB/c mouse experiments to investigate whether three different PD markers (the RS ratio, CFU, and 16S rRNA burden) provide the same information or measure different biological responses. We asked whether a combination of PD markers measuring different responses distinguishes between treatments better than any single PD marker. Finally, we evaluated the ability of different PD markers and combinations of markers to indicate the treatment-shortening activity of regimens.

## RESULTS

### RS ratio, CFU, and 16S rRNA burden each measure different biological responses.

**FIG 1:**
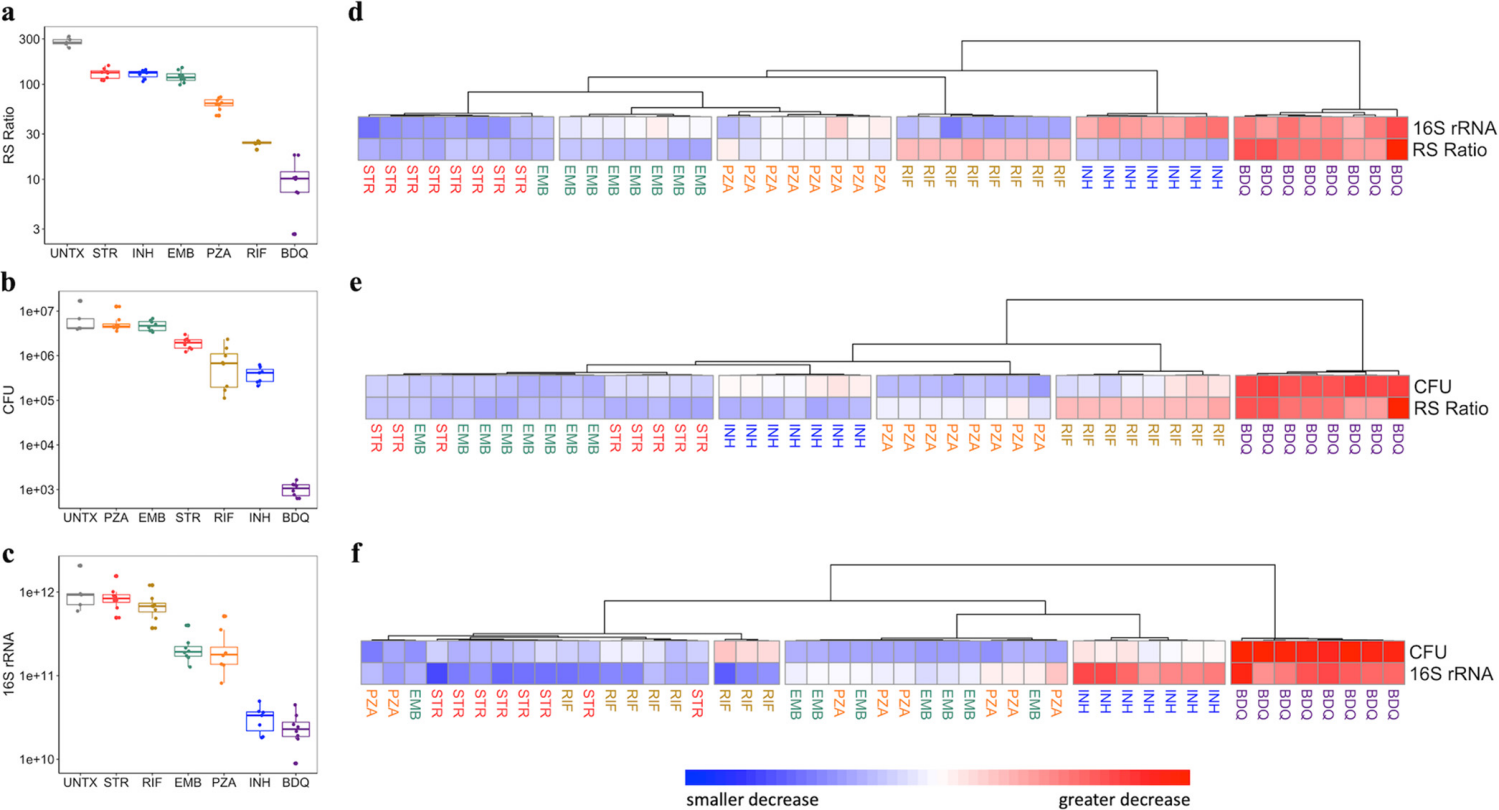
Effect of 4 weeks of treatment 5 of 7 days per week with individual drugs on different PD markers in the BALB/c mouse high-dose aerosol model. Individual drugs had differing effects on the RS ratio (a), CFU (b), and 16S rRNA burden (c). Points and boxes represent untreated control (gray), streptomycin (red), isoniazid (blue), ethambutol (green), pyrazinamide (orange), rifampin (golden), and bedaquiline (purple). Error bars indicate standard deviations. Hierarchical clustering shows drug effect on 16S rRNA burden and the RS ratio (d), CFU and the RS ratio (e), and CFU and 16S rRNA burden (f) in BALB/c mice. Hierarchical clustering was performed using pheatmap in R with the “Ward.D” agglomeration and “Euclidean” distance methods. Rows represent individual PD markers. Columns represent individual mice. Cell values represent a log_10_ decrease relative to control. Red, white and blue colors indicate greater, average, and smaller decreases, respectively. *n* = 8 mice in each treatment group except for untreated control (*n* = 5). Mice were treated with bedaquiline (BDQ), ethambutol (EMB), isoniazid (INH), pyrazinamide (PZA), rifampin (RIF) and streptomycin (STR). A group of untreated mice (UNTX) served as control group. One mouse in the INH group was euthanized (day 18) due to clinical disease resulting in its removal from the analysis.

Treatment with individual drugs affected each of the three PD markers differently (Fig. 1a to c), suggesting that each PD marker measures distinct biological responses. For example, rifampin and isoniazid had similar effects on CFU (*P = *0.46), but rifampin suppressed the RS ratio 6-fold more than isoniazid (*P = *0.0003). Conversely, isoniazid suppressed 16S rRNA burden 25-fold more than rifampin (*P = *0.0003). Although both CFU and 16S rRNA burden aim to enumerate the quantity of M. tuberculosis, they did not provide identical information. For example, the effects of isoniazid and bedaquiline on 16S rRNA burden were indistinguishable (*P = *0.3), but bedaquiline reduced CFU 400-fold more than isoniazid (*P = *0.001). Log_10_ decreases and *P* values for all drugs and all PD markers are included in Table S1 in the supplemental material.

Each PD marker assessed the rank order of drugs effect differently. For example, isoniazid had the second greatest effect on both CFU and 16S rRNA burden (Fig. 1b and c) but tied with streptomycin for the least effect on the RS ratio (Fig. 1a). Pyrazinamide tied with ethambutol for the least effect on CFU (Fig. 1b) but had the third greatest effect on 16S rRNA burden (Fig. 1c) and the RS ratio (Fig. 1a).

### Pairwise combinations of PD markers that include the RS ratio distinguish between drugs better than any individual PD marker.

Although no single PD marker was capable of distinguishing between all individual drugs, the distinct effect of drugs could be resolved based on combinations of PD markers that included the RS ratio. Hierarchical clustering demonstrated that the combination of the RS Ratio and 16S rRNA burden differentiated each drug from every other drug (Fig. 1d). Similarly, the combination of the RS ratio and CFU differentiated between all drugs with the exception that streptomycin and ethambutol grouped together (Fig. 1e). By contrast, the combination of CFU and 16S rRNA burden largely failed to distinguish between drugs (Fig. 1f). Only isoniazid and bedaquiline were clearly distinguishable; other drugs could not be differentiated based on the combination of CFU and 16S rRNA burden.

### Rank order of treatment-shortening activity in BALB/c relapsing mouse experiments.

Experiments 2 and 3 quantified treatment-shortening activity of combination drug regimens in the BALB/c relapsing TB mouse models based on the conventional microbiologic relapse outcome. The relapse outcomes are summarized in Table S2 in the supplemental material. Table 1 summarizes treatment duration that results in 95% cure (*T*_95_) values and the relapse rank order. In experiment 2, the rank order of treatment-shortening activity based on microbiologic relapse was as follows: bedaquiline, moxifloxacin, pyrazinamide, rifabutin (BMZRb) (fastest) > bedaquiline, moxifloxacin, pyrazinamide (BMZ) > rifapentine, moxifloxacin, pyrazinamide (PMZ) > isoniazid, rifampin, pyrazinamide, ethambutol (HRZE) (slowest) (11). In experiment 3, the rank order of treatment-shortening activity based on microbiologic relapse was as follows: bedaquiline, pretomanid, moxifloxacin, pyrazinamide (BPaMZ) (fastest) > bedaquiline, pretomanid, linezolid (BPaL) > pretomanid, moxifloxacin, pyrazinamide (PaMZ) > HRZE (slowest) (10). The sigmoidal maximum effect (*E*_max_) model improved model fit and detected significant differences between treatment regimens compared to the hyperbolic *E*_max_ model (γ = 1) (see Fig. S1 in the supplemental material).

**TABLE 1:** Treatment-shortening activity of diverse regimens in two BALB/c TB relapsing mouse model experiments based on the conventional microbiologic relapse outcome^*a*^

Expt and regimen	*T*_95_ in wks (95% confidence interval)	Rank of treatment-shortening activity
Expt 2		
Bedaquiline, moxifloxacin, pyrazinamide, rifabutin (BMZRb)	6.10 (5.68, 6.31)	1
Bedaquiline, moxifloxacin, pyrazinamide (BMZ)	7.09 (7.05, 7.28)	2
Rifapentine, moxifloxacin, pyrazinamide (PMZ)	8.03 (7.64, 9.82)	3
Isoniazid, rifampin, pyrazinamide, ethambutol (HRZE)	18.63 (18.44, 18.90)	4
Expt 3		
Bedaquiline, pretomanid, moxifloxacin, pyrazinamide (BPaMZ)	5.59 (5.33, 6.13)	1
Bedaquiline, pretomanid, linezolid (BPaL)	10.00 (9.79, 10.30)	2
Pretomanid, moxifloxacin, pyrazinamide (PaMZ)	13.12 (12.06, 13.68)	3
Isoniazid, rifampin, pyrazinamide, ethambutol (HRZE)	21.21 (20.70, 21.78)	4

a Regimen composition, *T*_95_ and rank order of treatment-shortening activity are shown for Experiments 2 and 3.

### Correlation of individual PD markers with treatment-shortening rank order.

**FIG 2:**
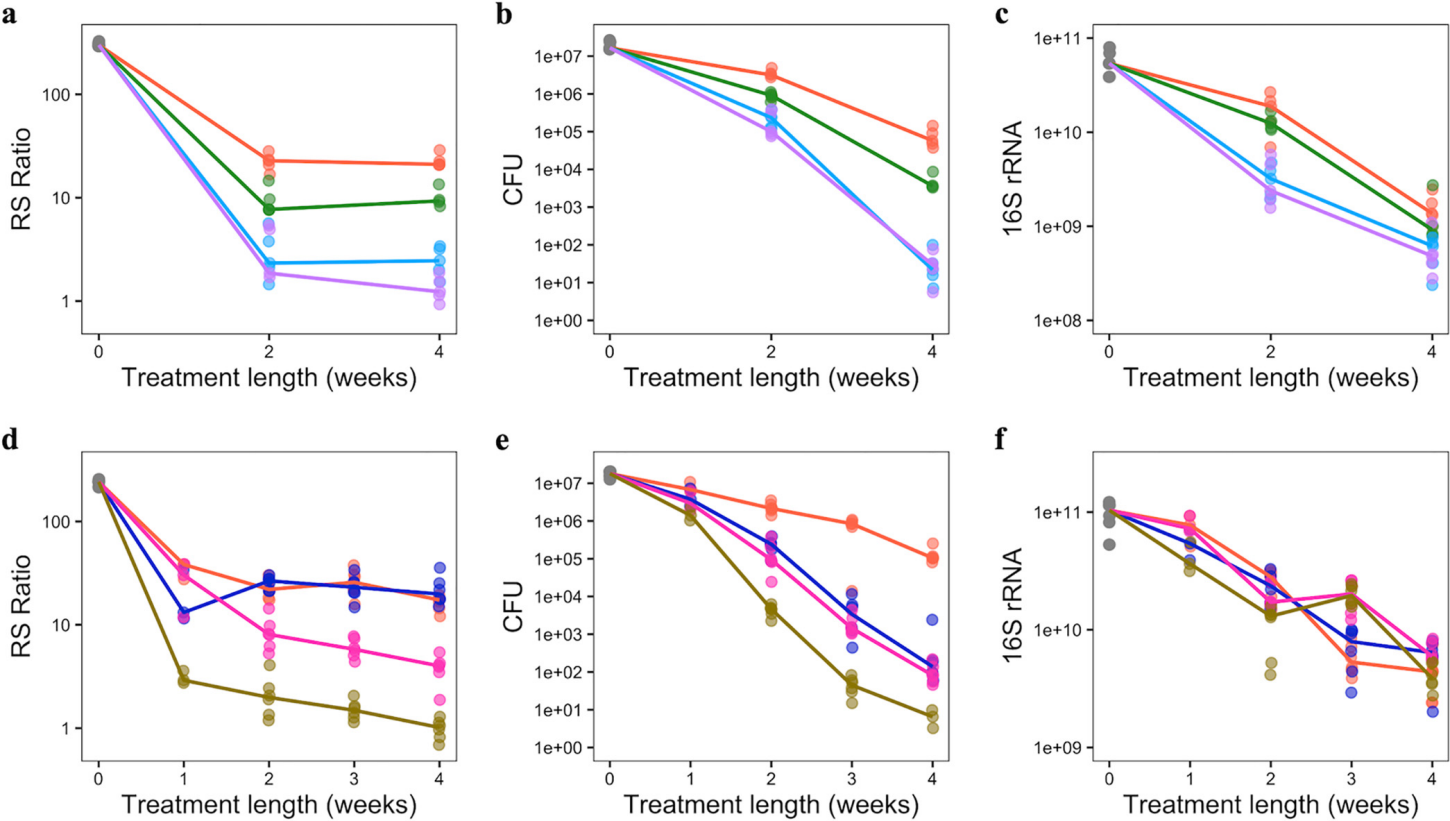
Differing effects of treatment on three PD markers during the first 4 weeks of treatment using data from two BALB/c TB relapsing mouse model experiments. RS ratio, CFU, and 16S rRNA burden were measured in lung homogenate of untreated mice (gray) and mice treated with BMZRb (purple), BMZ (light blue), PMZ (green), and HRZE (orange) in experiment 2 (a, b, c) and with BPaMZ (golden), BPaL (pink), PaMZ (blue), and HRZE in experiment 3 (d, e, f). Dots represent values from individual mice. Solid lines connect median values. All graphics use a log_10_ scale for the *y* axis. The control and treatment regimens each had 5 mice (experiment 2) and 6 mice (experiment 3).

**FIG 3:**
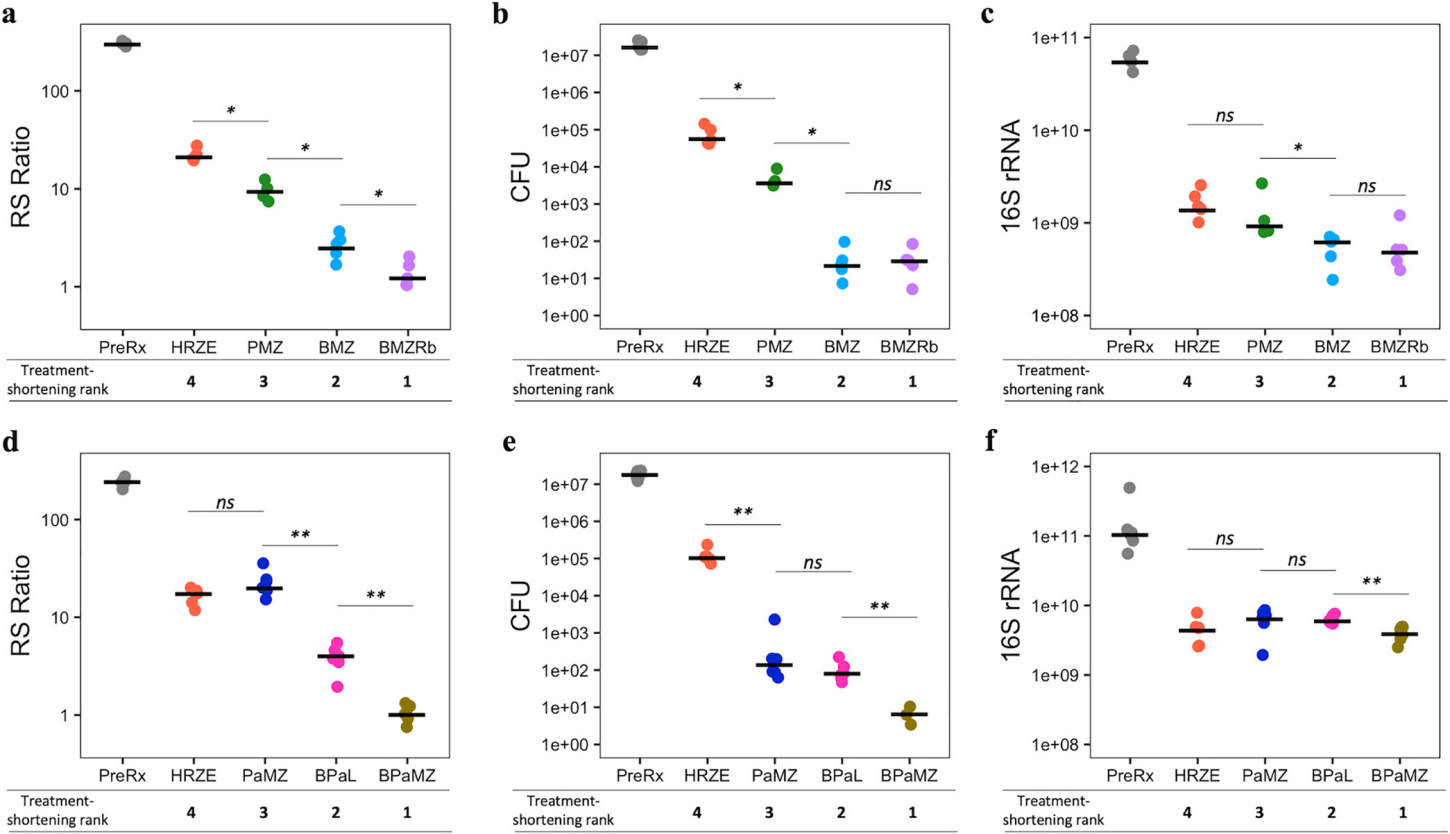
Correlation of RS ratio, CFU, and 16S rRNA burden with treatment-shortening rank order after 4 weeks of treatment using data from two BALB/c TB relapsing mouse model experiments. RS ratio, CFU, and 16S rRNA burden were measured in lung homogenate of untreated mice (gray) and mice treated with BMZRb (purple), BMZ (light blue), PMZ (green), and HRZE (orange) in experiment 2 (a, b, c) and with BPaMZ (golden), BPaL (pink), PaMZ (blue), and HRZE in experiment 3 (d, e, f). Dots represent values from individual mice. Bars represent median values. *P* value symbols are as follows: ns, nonsignificant; *, *P* value < 0.05; **, *P* value < 0.01. All graphics use a log_10_ scale for the *y* axis.

Consistent with experiment 1, treatment with combination regimens in experiments 2 and 3 affected the RS ratio, CFU, and 16S rRNA burden differently, confirming that they measure distinct biological responses (Fig. 2a to f). Individually, the three PD markers had variable ability to recapitulate the rank order of treatment-shortening activity of regimens (Fig. 3). After only 4 weeks of treatment in experiment 2, the RS ratio by itself matched the rank order of treatment-shortening activity measured many months later (Fig. 3a). CFU by itself did not distinguish the first ranked regimen (BMZRb) from the second ranked regimen (BMZ) (Fig. 3b). The 16S rRNA burden by itself did not match the treatment-shortening rank order except that it distinguished between the second (BMZ) and third (PMZ) ranked regimens (Fig. 3c). After 4 weeks of treatment in experiment 3, the RS ratio alone did not distinguish between the third (PaMZ) and fourth (HRZE) ranked regimens (Fig. 3d). Likewise, CFU alone did not distinguish between the second (BPaL) and third (PaMZ) ranked regimens (Fig. 3e). Again, 16S rRNA burden alone largely failed to distinguish between treatment regimens (Fig. 3f). Table S3 in the supplemental material summarizes the correlation of individual PD markers with rank order of regimens at the earliest time points for both experiments. Table S4 in the supplemental material includes log_10_ decreases for all treatment regimens and time points.

### Combination of the RS ratio and CFU improves distinction and classification of treatment regimens.

**FIG 4:**
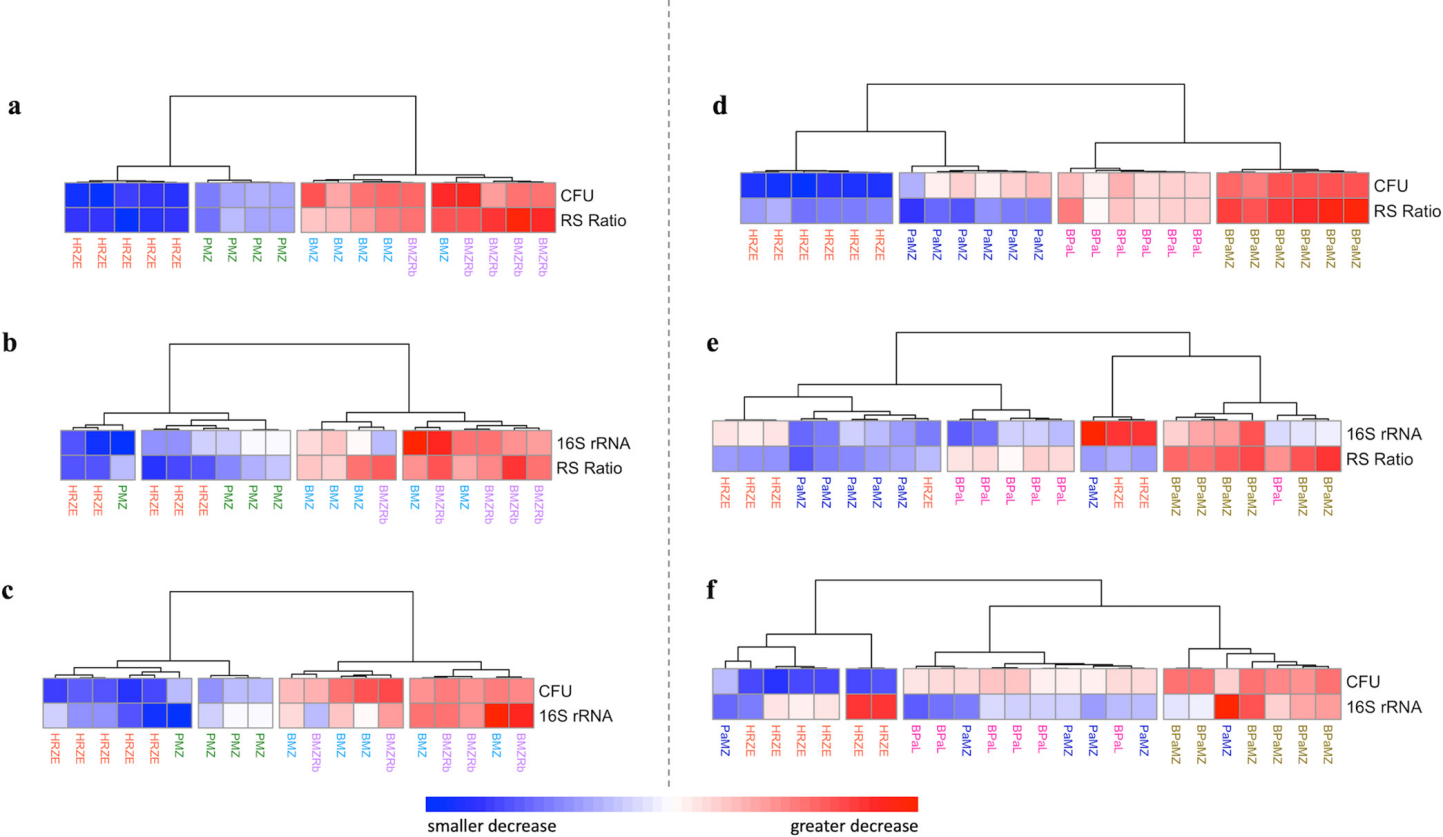
Distinguishing different regimens based on three PD markers measured after 4 weeks of treatment using data from two BALB/c TB relapsing mouse model experiments. Combination of PD markers are shown for experiment 2 (a, b, c) and experiment 3 (d, e, f). Hierarchical clustering was performed using pheatmap in R with the “Ward.D” agglomeration and “Euclidean” distance methods. Rows represent individual PD markers. Columns represent individual mice. Cell values represent log_10_ decrease relative to control. Red, white, and blue colors indicate greater, average, and smaller decreases, respectively. The control and treatment regimens each had 5 mice (experiment 2) and 6 mice (experiment 3). RS ratio could not be quantified in one mouse treated with PMZ in experiment 2 resulting in its removal from the analysis.

Combining different types of PD markers assisted in distinguishing the distinct effects of different regimens. After 4 weeks of treatment, the combination of the RS ratio with CFU near-perfectly distinguished between regimens in experiment 2 (Fig. 4a) and perfectly distinguished between regimens in experiment 3 (Fig. 4d). The degree to which regimens decreased CFU and RS ratio appeared concordant with treatment-shortening activity (Fig. 4a and d). By contrast, the combination of 16S rRNA burden with either the RS ratio (Fig. 4b and e) or CFU (Fig. 4c and f) failed to distinguish between treatment regimens.

## DISCUSSION

Our analysis of three BALB/c mouse experiments, conducted at two different institutions using different infecting strains, demonstrated that the RS ratio, CFU, and 16S rRNA burden each measure different biological responses to drug treatment. The RS ratio is a nonculture-based assay that provides orthogonal information and correlates with regimen treatment-shortening activity. Combining different PD markers enhanced distinction between treatments, relative to any single marker alone. The combination of the RS ratio with CFU showed the greatest ability to recapitulate the rank order of regimens, providing proof of concept that assessment of regimen treatment-shortening activity within the first weeks of treatment may be possible. Development of an early accurate measure of treatment-shortening activity has potential to transform the design of murine efficacy studies, thereby accelerating evaluation of new more potent regimens.

CFU has been a standard historical marker in murine studies despite widely recognized limitations. Our results reinforce previous evidence that change in lung CFU during the first weeks of treatment in mice does not indicate treatment-shortening activity (1). Perhaps relatedly, McCune and colleagues in the 1950s (12, 13) and more contemporary investigators (14–19) have shown that CFU quantifies only the subset of the M. tuberculosis population that is capable of growth on solid medium and often does not detect the last remaining viable bacilli that determine the treatment duration necessary to prevent relapse in mice. These limitations highlight the potential value of gathering alternative information from murine drug studies and motivated our development of molecular measures of treatment effect.

rRNA has been proposed as a means of enumerating the entire M. tuberculosis population, including subpopulations capable and incapable of colony formation on solid media (6). de Knegt et al. (6) previously described a striking divergence between reduction in CFU and reduction in the molecular bacterial load assay (MBLA) (a measure of rRNA burden) in BALB/c mice. For example, de Knegt found that, after 8 to 12 weeks of treatment with HRZE, CFU decreased >100-fold more than MBLA. Our current experiments demonstrated a similar disconnect in which CFU decreased more than 16S rRNA burden. It remains unclear whether the sustained high rRNA burden during treatment indicates the presence of a continuing large nonculturable M. tuberculosis population or detection of residual rRNA from dead M. tuberculosis. Our experiments confirmed de Knegt’s observation that change in rRNA burden largely fails to distinguish between regimens with different treatment-shortening potency in mice.

Unlike CFU and rRNA, which estimate bacterial burden, the RS ratio was designed to measure an alternative property, the degree to which drugs and regimens interrupt rRNA synthesis. Each of our three experiments demonstrated that the RS ratio provides information that is orthogonal to CFU or rRNA burden. Experiment 2 showed that the RS ratio was able to measure the effect of adding single drug (Rb) to a potent combination (BMZ), a difference that was not identifiable based on CFU. Change in the RS ratio correlated with the treatment-shortening activity of regimens. These observations suggest that the RS ratio may be a valuable new nonculture-based tool for preclinical efficacy evaluation.

This study also provides proof of concept that different readouts of drug effect (i.e., CFU and RS ratio) can be complementary, and their combination may be more informative than either PD marker alone. A next step will be development of a composite outcome incorporating CFU and the RS ratio to improve early efficacy assessment in mice. This would require a development phase in which both CFU and the RS ratio are collected in additional relapsing mouse trials testing diverse regimens. These results would enable parameterization of a composite outcome and evaluation of the composite CFU-RS ratio (quantified during the first treatment weeks of treatment) as a surrogate for subsequent relapse. If prediction of relapse is validated, a composite CFU-RS ratio assay would enable higher throughput murine screening studies in which a large number of regimens is tested in 1-month studies to “funnel down” to top candidates that can then proceed to traditional, lengthy, resource-intensive, relapsing TB mouse model experiments. Availability of a method that reliably predicts treatment-shortening efficacy based on responses during the first weeks of treatment would alleviate a key bottleneck in the preclinical TB drug evaluation process.

This study has several limitations. First, an inherent challenge to evaluating the predictive value of PD markers in mice is that mice can only be sacrificed once. Because it is not possible to measure PD markers early in treatment and the relapse outcome in the same individual mouse, predictive modeling is inherently limited. Second, as noted above, this report demonstrates proof of concept based on two relapsing mouse studies, establishing a starting point. Parameterization and validation of a composite CFU-RS ratio will require additional relapse studies with more diverse regimens. Finally, the estimated *T*_95_ for HRZE differed between experiment 2 conducted at Johns Hopkins University (JHU) and experiment 3 conducted at Colorado State University (CSU). This difference may be attributable to the different M. tuberculosis strains used at JHU and CSU or another experimental factor (20).

In summary, this analysis highlights the potential to harness multiple different types of PD markers to extract greater insight from animal models and accelerate development of new combination regimens. New molecular tools like the RS ratio offer potential for a new era in which antimicrobial therapies are evaluated not only on culture-based measures of bacterial burden but also on molecular assays that indicate how drugs impact the physiological state of the pathogen.

## MATERIALS AND METHODS

We compared three PD markers in three BALB/c mouse experiments in two labs. Experiment 1 evaluated individual drugs to determine whether the RS ratio, CFU/lung, and 16S rRNA burden measure the same or different biological responses. Experiments 2 and 3 evaluated combination regimens to determine whether changes in PD markers during the first weeks of treatment distinguish between regimens and indicate regimen treatment-shortening activity. Efficacy outcomes from experiments 2 and 3 are reported elsewhere (10, 11).

### Description of BALB/c mouse experiments.

Full details of murine protocols are included in the supplemental material and in other publications (10, 11). Briefly, all three experiments employed female pathogen-free BALB/c mice infected by the same high-dose aerosol (HDA) procedure in a Glas-Col inhalation exposure system (21, 22) and treated mice 5 of 7 days a week via oral gavage. Experiments 1 and 3 were conducted at Colorado State University using the M. tuberculosis Erdman strain. Experiment 2 was conducted at Johns Hopkins University using the M. tuberculosis H37Rv strain.

### (i) Experiment 1: individual drug treatments in BALB/c mouse HDA infection model.

Treatment began on day 11 and continued for 4 weeks. The doses (in mg/kg of body weight indicated in subscripts) tested were as follows: bedaquiline (B_25_), ethambutol (E_100_), isoniazid (H_25_), pyrazinamide (Z_150_), rifampin (R_10_), and streptomycin (S_200_). Each treatment group had eight mice except for the untreated control, which had five mice.

### (ii) Experiments 2 and 3: multidrug treatments in BALB/c HDA relapsing mouse model.

Experiments 2 and 3 used the standard conventional relapsing mouse model described in the supplemental material (1, 10, 11). In experiment 2, treatment began on day 14 postinfection with the following: isoniazid, rifampin, pyrazinamide, ethambutol (HRZE); rifapentine, moxifloxacin, pyrazinamide (PMZ); bedaquiline, moxifloxacin, pyrazinamide (BMZ); or bedaquiline, moxifloxacin, pyrazinamide, rifabutin (BMZRb). In experiment 3, treatment began on day 11 with the following: HRZE using doses identical to experiment 2; pretomanid, moxifloxacin, pyrazinamide (PaMZ); bedaquiline, pretomanid, linezolid (BPaL); or bedaquiline, pretomanid, moxifloxacin, pyrazinamide (BPaMZ). The doses (in mg/kg indicated in subscripts) tested were as follows: H_10_, R_10_, Z_150_, E_100_, P_10_, M_100_, B_25_, Rb_10_, Pa_100_, and L_100_. The control and treatment regimens each had five mice in experiment 2, each separate from the mice used for CFU counts in the companion report. The control and treatment regimens each had six mice in experiment 3.

### Tissue collection.

Mice were euthanized the day after the final treatment dose one at a time via CO_2_ asphyxiation. Upper right lung lobes were flash frozen in liquid nitrogen for immediate RNA preservation and then homogenized and lysed via bead beating as described in the supplemental material. Remining lung lobes were collected for enumeration of CFU.

### Quantification of PD markers.

Following RNA extraction and reverse transcription to cDNA, TaqMan quantitative PCR (qPCR) was used to quantify abundance of 16S rRNA as described in the supplemental material. The RS ratio was determined in a duplex assay using the QX100 Droplet Digital PCR system (Bio-Rad) as described in the supplemental material. Primers and probe sequences are in the supplemental material. CFU burdens were estimated by serial dilutions of lung homogenates and plating on 7H11-oleic acid-albumin-dextrose-catalase (OADC) agar using 0.4% activated charcoal to prevent drug carryover as described in the supplemental material.

### Ethical approval and oversight.

Murine experiments were performed in certified animal biosafety level III facilities with appropriate institutional approvals as described in the supplemental material.

### Statistical analysis.

Two-sample Wilcoxon tests were used for pairwise comparisons. For experiments 2 and 3, a Bayesian sigmoidal *E*_max_ model was applied using the function “stan_emax” in the rstanemax R package to determine, for individual regimens, the treatment duration that results in 95% cure (*T*_95_). Then, *T*_95_ values were compared to establish a rank order of treatment-shortening activity. Lower *T*_95_ values indicate greater treatment-shortening activity. Description of the sigmoidal *E*_max_ model is included in the supplemental material.

Hierarchical clustering was used to evaluate the ability of combinations of PD markers to distinguish drugs and regimens. Differences were considered significant at the 95% confidence level. Analysis was conducted using R (v 3.5.3; R Development Core Team, Vienna, Austria).

### Data availability.

All primary data is included in the supplemental material.
