# Supplementary material for: Combination of Mycobacterium tuberculosis RS Ratio and CFU Improves the Ability of Murine Efficacy Experiments to Distinguish between Drug Treatments
Source: Antimicrob Agents Chemother. 2022 Mar 21;66(4):e02310-21. doi: 10.1128/aac.02310-21 (PMC9017352; doi:10.1128/aac.02310-21)
Supplement: Supplemental file 1 — Supplemental methods, Tables S1 to S4, and Fig. S1. Download aac.02310-21-s0001.pdf, PDF file, 0.4 MB [file aac.02310-21-s0001.pdf]

Combination of *Mycobacterium tuberculosis* RS ratio and CFU improves the ability of murine efficacy experiments to distinguish between drug treatments

Christian Dide-Agossou,[1†] Allison A. Bauman,[2] Michelle E. Ramey,[2] Karen Rossmassler,[3,4] Reem Al Mubarak,[3,4] Samantha Pauly,[3,4] Martin I. Voskuil,[5,6] Maria Garcia-Cremades,[7,8] Rada M. Savic,[6,7,9,10] Payam Nahid,[6,9,10,11] Camille M. Moore,[12] Rokeya Tasneen,[13] Eric L. Nuermberger,[13] Gregory T. Robertson,[2,6] Nicholas D. Walter[3,4,6]

† Corresponding author (CD-A)

Table of Contents

SUPPLEMENTAL METHODS 2

- Description of BALB/c mouse experiments 2
- RNA extraction from murine samples 3
- Quantification of rRNA burden via RT-qPCR 4
- Quantification of the RS Ratio via droplet digital PCR 4
- Enumeration of colony forming units (CFU) from lungs 4
- Description of the Emax model 5

SUPPLEMENTAL FIGURES & TABLES 6

- Table S1. Effect of individual drugs on RS Ratio, CFU and 16S rRNA in Experiment 1. 6
- Table S2. Relapse proportions for different treatment durations in Experiments 2 and 3. 7
- Figure S1. Hyperbolic and sigmoidal E_{max} models in BALB/c relapsing mouse experiments. 8
- Table S3. P-values for pairwise comparisons of RS Ratio, CFU and 16S rRNA burden in Experiments 2 and 3. 9
- Table S4. Log_{10} decrease in RS Ratio, CFU and 16S rRNA in Experiments 2 and 3. 10

Supplemental methods

Description of BALB/c mouse experiments

Experiment 1: BALB/c mouse high dose aerosol infection model

Six to 8-week-old female pathogen-free BALB/c mice (Jackson Laboratories) were exposed to high-dose aerosol of *M. tuberculosis* Erdman from broth culture (OD_{600} ~ 0.8) to achieve deposition of ~3.8 log10 CFU in the lungs of each mouse (1, 2). Treatment was initiated on day 11 post aerosol and continued for 4 (3) weeks. Groups of 6 mice were individually euthanized by CO_2 narcosis on day 11, prior to treatment initiation, and on the last day of treatment, to determine the bacterial loads in lungs. The left and lower right lung lobes (inferior and post-caval lobes) were used for bacterial enumeration. Upper right lung lobes (superior and middle lobes) were flash frozen in liquid nitrogen prior to RNA extraction.

Experiments 2 and 3: BALB/c mouse relapsing models

Six to 8-week-old female pathogen-free BALB/c mice (Jackson Laboratories) were exposed to high-dose aerosol of *M. tuberculosis* from broth culture (OD_{600} ~0.8) to achieve deposition of ~ 4.3 log_{10} CFU in the lungs of each mouse (1, 2). Treatment was initiated on post-infection day 15 (Experiment 2 at JHU) or day 11 (Experiment 3 at CSU) and continued for up to 20 weeks. Groups of 3 to 6 mice, as indicated, were individually euthanized by CO_2 narcosis prior to treatment initiation, and one day following the last day of treatment, to determine the bacterial loads in the lungs. Additional groups of 15 mice each from each treatment group were placed on a 12 week drug holiday for evaluation of the conventional microbiological relapse outcome (1, 4–7). The left and lower right lung lobes (inferior and post-caval lobes) were used for bacterial enumeration. Upper right lung lobes (superior and middle lobes) were flash frozen in liquid nitrogen prior to beadbeating and RNA extraction.

RNA extraction from murine samples

Lung tissues from murine samples were bead beaten in 2 ml Trizol using the CKmix50 lysis kit for 1×16 seconds at 7,200 rpm followed by 3×30 seconds at 6,500 rpm with 5 minute rests on wet ice between cycles using the CK01 lysis kit (Bertin). Cellular debris was separated from the cell lysate by centrifugation for 1 minute at 21,000×g. Trizol lysates were transferred to a new tube containing heavy phase lock gel and 300 µl chloroform, mixed vigorously for 15 seconds, then incubated at room temperature for 2 minutes with occasional mixing followed by centrifugation at 21,000×g for 10 minutes. The aqueous phase was removed to a separate tube for RNA extraction and mixed with 270 µl of isopropanol. 265 µl of high salt solution (0.8 M sodium citrate, 1.2 M sodium chloride) was added and mixed by inversion. RNA was precipitated overnight at 4°C and then pelleted by centrifugation at 4°C, 21,000×g for 10 minutes. The RNA pellet was air dried for 10 minutes at room temperature, resuspended in 80 µl water, and reconstituted for one hour on ice. 2 µl DNase I and 10 µl DNase I buffer (Promega) and 2.5 µl Ribolock RNase inhibitor were added to the resuspended pellet and incubated for 30 minutes at 37°C. RNA was then purified with the Maxwell RSC simplyRNA tissue kit using the Maxwell RSC instrument (Promega) following the manufacturer's instructions with the following modifications: an additional DNase treatment was performed with Promega RQ1 DNase prior to instrument use and additional kit DNase was added at twice the recommended amount for instrument use. For every extraction batch, a negative extraction control was included to test for cross-contamination between samples. RNA extraction efficiency was tracked by spiking in a commercially available external RNA control mix (ERCC) (Ambion, # 4456740) immediately prior to extraction. The RNA extraction efficiency was calculated as a percentage of RNA that was retained during the extraction process. To track RNA retention percentage, an

equal volume of the ERCC mix from the same stock was diluted to match the final RNA elution volume.

Quantification of rRNA burden via RT-qPCR

After extraction, RNA was reverse transcribed with SuperScript VILO cDNA synthesis kit (Invitrogen) for 120 min at 42°C following the manufacturer's protocol. The resulting cDNA was diluted to 1:10. Then, 4 µl of the diluted cDNA was used as input in a RT-qPCR step (TaqMan assay) for the absolute quantification of 16S. To determine the retention percentage, two templates in the ERCC mix were targeted by a duplexed RT-qPCR (ERCC002 and ERCC130). Primers and probe sequences and thermocycling conditions were as previously described (3). RNA retention percentage was calculated by taking the inverse of the fold change between the Ct values of the sample and each ERCC control. The average RNA retention percentage was used in rRNA burden calculation. *M. tuberculosis* rRNA burden was expressed as the absolute count of 16S rRNA gene transcripts adjusted by the RNA retention percentage and dilution factors.

Quantification of the RS Ratio via droplet digital PCR

RNA was reverse transcribed with SuperScript VILO to cDNA as described above. 2 µl RNA was used for a 10 µl reaction. Transcript copies were quantified using the QX100 Droplet Digital PCR system (Bio-Rad). Reaction were run in duplex (*i.e.,* ETS1 with 23S) with ddPCR SuperMix for Probes (no dUTP) (Bio-Rad). The ratio of ETS1/23S was calculated within each duplexed reaction by QX100 Droplet Digital PCR system software (Bio-Rad).

Enumeration of colony forming units (CFU) from lungs

For Experiment 2 at Johns Hopkins University, enumeration of CFU is described elsewhere (8). For Experiment 3, the number of viable organisms was determined by serial

dilutions of homogenates (Precellys Evolution, Bertin) prepared in phosphate buffered saline plus 10% (w/v) bovine serum albumin from indicated lung lobes and plating on 7H11-OADC agar plates containing 0.4% (w/v) activated charcoal to prevent drug carry-over. Colonies were enumerated after at least 21 days of incubation at 37°C. For relapse assessments, tissues were homogenized in PBS and plated in their entirety on 7H11-OADC agar plates without activated charcoal.

Description of the Emax model

The sigmoidal E_{max} model was tested according to:

$$P_{cure} = \left(1 - P_{relapse}\right) = E_0 + \frac{E_{max} \times T^{\gamma}}{T_{50}^{\gamma} + T^{\gamma}}$$

In this model, P_{cure} is the probability of cure defined as a negative solid culture 3 months post-treatment. The independent variable is treatment length. E_0 is the expected response when exposure is zero (*i.e.*, basal effect). E_{max} is the maximal achievable probability of cure. The E_0 and E_{max} parameters were constrained to 0 and 1, respectively. γ is a shape parameter controlling the steepness of the curve produced by the E_{max} equation. T_{50} is the treatment length at which half the E_{max} is achieved. From the T_{50} estimates we derived T_{95} of the combination regimens according to the formula:

$$T_{95} = T_{50} \times \left(\frac{95}{100 - 95}\right)^{1/\gamma}$$

SUPPLEMENTAL FIGURES & TABLES

Table S1. Effect of individual drugs on RS Ratio, CFU and 16S rRNA in Experiment 1. Log_{10} decrease relative to untreated control and pairwise comparison *P*-values between mice treated with pyrazinamide (PZA), ethambutol (EMB), streptomycin (STR), rifampin (RIF), isoniazid (INH) and bedaquiline (BDQ). Pairwise comparisons between individual drugs were performed using two-sample Wilcoxon tests. Average log_{10} decrease are shown in the top row and left-hand column. *P*-values are shown in black. Statistically significant *P*-values are highlighted in bold.

RS Ratio

		PZA	EMB	STR	RIF	INH	BDQ
	log_{10} decrease	*0.6*	*0.4*	*0.3*	*1.1*	*0.3*	*1.4*
PZA	*0.6*		**0.0002**	**0.0002**	**0.0002**	**0.0003**	**0.0002**
EMB	*0.4*			0.4	**0.0002**	0.5	**0.0002**
STR	*0.3*				**0.0002**	0.8	**0.0002**
RIF	*1.1*					**0.0003**	**0.0002**
INH	*0.3*						**0.0003**
BDQ	*1.4*						

CFU

		PZA	EMB	STR	RIF	INH	BDQ
	log_{10} decrease	*0.2*	*0.2*	*0.6*	*1.0*	*1.2*	*3.8*
PZA	*0.2*		0.8	**0.0002**	**0.0002**	**0.0003**	**0.0009**
EMB	*0.2*			**0.0009**	**0.0009**	**0.001**	**0.0009**
STR	*0.6*				0.02	**0.0003**	**0.0009**
RIF	*1.0*					0.45	**0.0009**
INH	*1.2*						**0.001**
BDQ	*3.8*						

16S rRNA

		PZA	EMB	STR	RIF	INH	BDQ
	log_{10} decrease	*0.8*	*0.7*	*0.1*	*0.2*	*1.5*	*1.7*
PZA	*0.8*		0.6	**0.0003**	**0.0006**	**0.0003**	**0.0002**
EMB	*0.7*			**0.0002**	**0.0003**	**0.0003**	**0.0002**
STR	*0.1*				0.2	**0.0003**	**0.0002**
RIF	*0.2*					**0.0003**	**0.0002**
INH	*1.5*						0.3
BDQ	*1.7*						

Table S2. Relapse proportions for different treatment durations in Experiments 2 and 3.

Experiment 2: Johns Hopkins University

Treatment Weeks (16 14 12 10 8 6 4)	Relapse Assessment: Relapse outcomes	Relapse Assessment: Relapse rates
BMZRb	15/15	100%
BMZRb	1/15	7%
BMZ	15/15	100%
BMZ	9/15	60%
PMZ	13/14	93%
PMZ	0/15	0%
2HRZE/3HR	15/15	100%
2HRZE/3HR	8/15	53%

Experiment 3: Colorado State University

Treatment Weeks (20 16 12 8 4)	Relapse Assessment: Relapse outcomes	Relapse Assessment: Relapse rates
BPaMZ	13/15	87%
BPaMZ	1/15	7%
BPaMZ	0/15	0%
BPaL	15/15	100%
BPaL	9/15	60%
BPaL	0/15	0%
PaMZ	3/15	20%
PaMZ	0/15	0%
PaMZ	0/15	0%
2HRZE/3HR	15/15	100%
2HRZE/3HR	12/15	80%
2HRZE/3HR	1/14	7%

	Hyperbolic E_{max} model	Sigmoidal E_{max} model
Experiment 2	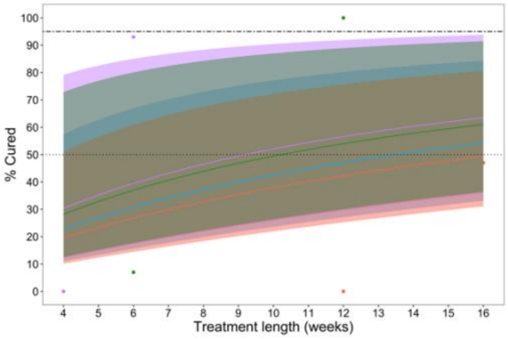	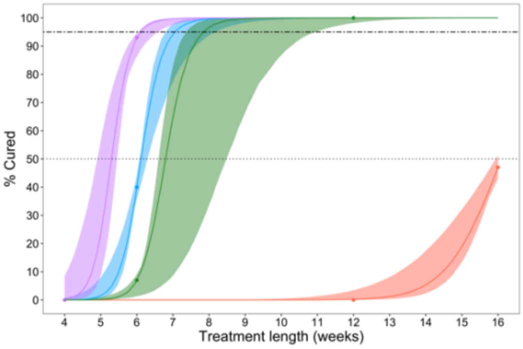
Experiment 3	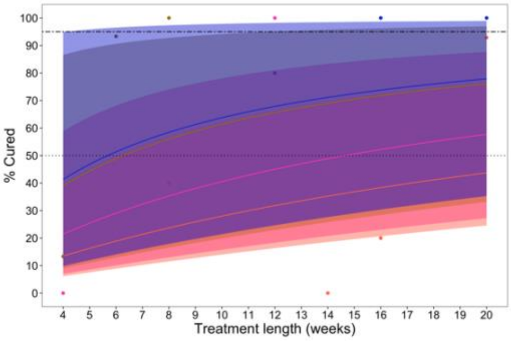	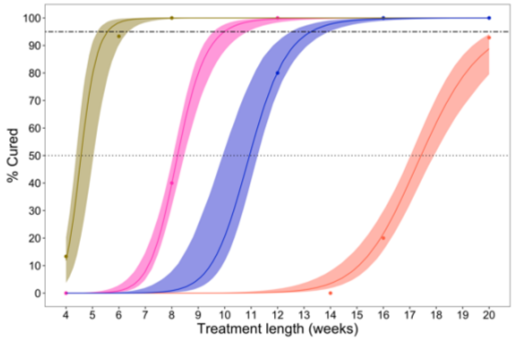

Figure S1. Hyperbolic and sigmoidal E_{max} models in BALB/c relapsing mouse experiments. E_{max} curves are shown for individual regimens and were derived from modeling the observed cure rates as function of treatment length using a Bayesian approach. The difference in expected predictive accuracy between the hyperbolic and sigmoidal E_{max} models was tested using the leave-one-out cross-validation method.(9) Observed cure rates (dots) and predicted cure rates (solid line) with 95% credible interval range (shaded area) are shown for BMZRb (purple), BMZ (light blue), PMZ (green) and HRZE (orange) in Experiment 2, and for BPaMZ (golden), BPaL (pink), PaMZ (blue) and HRZE in Experiment 3. Dotted and dashed lines represent T_{50} and T_{95}, respectively. N=15 mice in each treatment group.

Table S3. *P*-values for pairwise comparisons of RS Ratio, CFU and 16S rRNA burden in Experiments 2 and 3.

The rank order of regimen treatment shortening activity as estimated based on sigmoidal E_{max} models is also shown. Pairwise comparisons between regimens were performed using two-sample Wilcoxon tests. Statically significant *P*-values are highlighted in bold.

Experiment timepoint		RS Ratio			CFU			16S rRNA burden		
Experiment 2	Regimen (rank)	BMZ (*2nd*)	PMZ (*3rd*)	HRZE (*4th*)	BMZ (*2nd*)	PMZ (*3rd*)	HRZE (*4th*)	BMZ (*2nd*)	PMZ (*3rd*)	HRZE (*4th*)
W02	BMZRb (*1st*)	0.7			0.5			1.0		
	BMZ (*2nd*)		**0.008**			**0.008**			**0.008**	
	PMZ (*3rd*)			**0.008**			0.06			0.3
W04	BMZRb (*1st*)	**0.03**			0.9			1.0		
	BMZ (*2nd*)		**0.02**			**0.02**			**0.02**	
	PMZ (*3rd*)			**0.02**			**0.02**			0.3
Experiment 3	Regimen (rank)	BPaL (*2nd*)	PaMZ (*3rd*)	HRZE (*4th*)	BPaL (*2nd*)	PaMZ (*3rd*)	HRZE (*4th*)	BPaL (*2nd*)	PaMZ (*3rd*)	HRZE (*4th*)
W01	BPaMZ (*1st*)	0.1			0.2			0.1		
	BPaL (*2nd*)		0.7			0.7			0.2	
	PaMZ (*3rd*)			0.2			0.4			0.4
W02	BPaMZ (*1st*)	**0.002**			**0.002**			**0.002**		
	BPaL (*2nd*)		**0.002**			0.1			0.5	
	PaMZ (*3rd*)			0.3			**0.002**			0.8
W03	BPaMZ (*1st*)	**0.002**			**0.002**			0.9		
	BPaL (*2nd*)		**0.002**			0.4			**0.002**	
	PaMZ (*3rd*)			0.5			**0.005**			0.5
W04	BPaMZ (*1st*)	**0.002**			**0.004**			**0.002**		
	BPaL (*2nd*)		**0.002**			0.2			0.9	
	PaMZ (*3rd*)			0.2			**0.005**			0.2

Table S4. Log$_{10}$ decrease in RS Ratio, CFU and 16S rRNA in Experiments 2 and 3. Median log$_{10}$ decrease relative to untreated control for individual PD markers at various time points in BALB/c relapsing mouse experiments.

Experiment time point	Regimen	RS Ratio	CFU	16S rRNA burden
Experiment 2				
W02	BMZRb	*2.2*	*2.3*	*1.4*
	BMZ	*2.1*	*1.9*	*1.3*
	PMZ	*1.6*	*1.3*	*0.7*
	HRZE	*1.1*	*0.8*	*0.5*
W04	BMZRb	*2.4*	*5.8*	*2.1*
	BMZ	*2.1*	*5.9*	*2.0*
	PMZ	*1.5*	*3.7*	*1.8*
	HRZE	*1.2*	*2.5*	*1.6*
Experiment 3				
W01	BPaMZ	*1.9*	*1.1*	*0.6*
	BPaL	*0.9*	*0.8*	*0.3*
	PaMZ	*1.3*	*0.7*	*0.5*
	HRZE	*0.8*	*0.4*	*0.3*
W02	BPaMZ	*2.1*	*3.6*	*1.1*
	BPaL	*1.5*	*2.3*	*1.0*
	PaMZ	*0.9*	*1.9*	*0.8*
	HRZE	*1.0*	*0.9*	*0.7*
W03	BPaMZ	*2.2*	*5.6*	*0.9*
	BPaL	*1.6*	*4.1*	*0.9*
	PaMZ	*1.0*	*3.8*	*1.3*
	HRZE	*1.0*	*1.3*	*1.5*
W04	BPaMZ	*2.4*	*6.7*	*1.6*
	BPaL	*1.8*	*5.3*	*1.4*
	PaMZ	*1.1*	*5.1*	*1.4*
	HRZE	*1.1*	*2.2*	*1.5*

REFERENCES

1. Nuermberger E, Rosenthal I, Tyagi S, Williams KN, Almeida D, Peloquin CA, Bishai WR, Grosset JH. 2006. Combination chemotherapy with the nitroimidazopyran PA-824 and first-line drugs in a murine model of tuberculosis. Antimicrob Agents Chemother 50:2621–2625.
2. De Groote MA, Gilliland JC, Wells CL, Brooks EJ, Woolhiser LK, Gruppo V, Peloquin CA, Orme IM, Lenaerts AJ. 2011. Comparative studies evaluating mouse models used for efficacy testing of experimental drugs against Mycobacterium tuberculosis. Antimicrob Agents Chemother 55:1237–1247.
3. Walter ND, Born SEM, Robertson GT, Reichlen M, Dide-Agossou C, Ektnitphong VA, Rossmassler K, Ramey ME, Bauman AA, Ozols V, Bearrows SC, Schoolnik G, Dolganov G, Garcia B, Musisi E, Worodria W, Huang L, Davis JL, Nguyen NV, Nguyen HV, Nguyen ATV, Phan H, Wilusz C, Podell BK, Sanoussi ND, de Jong BC, Merle CS, Affolabi D, McIlleron H, Garcia-Cremades M, Maidji E, Eshun-Wilson F, Aguilar-Rodriguez B, Karthikeyan D, Mdluli K, Bansbach C, Lenaerts AJ, Savic RM, Nahid P, Vásquez JJ, Voskuil MI. 2021. Mycobacterium tuberculosis</i> precursor rRNA as a measure of treatment-shortening activity of drugs and regimens. Nat Commun 12:2899.
4. Li SY, Irwin SM, Converse PJ, Mdluli KE, Lenaerts AJ, Nuermberger EL. 2015. Evaluation of moxifloxacin-containing regimens in pathologically distinct murine tuberculosis models. Antimicrob Agents Chemother 59:4026–30.
5. Ammerman NC, Swanson RV, Bautista EM, Almeida DV, Saini V, Omansen TF, Guo H, Chang YS, Li S-Y, Tapley A, Tasneen R, Tyagi S, Betoudji F, Moodley C, Ngcobo B, Pillay L, Bester LA, Singh SD, Chaisson RE, Nuermberger E, Grosset JH. 2018. Impact of clofazimine dosing on treatment shortening of the first-line regimen in a mouse model of tuberculosis. Antimicrob Agents Chemother 62:e00636-18.
6. Saini V, Ammerman NC, Chang YS, Tasneen R, Chaisson RE, Jain S, Nuermberger E, Grosset JH. 2019. Treatment-shortening effect of a novel regimen combining clofazimine and high-dose rifapentine in pathologically distinct mouse models of tuberculosis. Antimicrob Agents Chemother 63:e00388-19.
7. Tasneen R, Betoudji F, Tyagi S, Li S-Y, Williams K, Converse PJ, Dartois V, Yang T, Mendel CM, Mdluli KE, Nuermberger EL. 2015. Contribution of oxazolidinones to the efficacy of novel regimens containing bedaquiline and pretomanid in a mouse model of tuberculosis. Antimicrob Agents Chemother 60:270–277.
8. Tasneen R, Garcia A, Converse PJ, Zimmerman MD, Dartois V, Kurbatova E, Vernon AA, Carr W, Stout JE, Dooley KE, Nuermberger EL. Novel regimens of bedaquiline-pyrazinamide combined with moxifloxacin, rifabutin, delamanid and/or OPC-167832 in murine tuberculosis models. Antimicrob Agents Chemother SUBMITTED AS COMPANION REPORT.
9. Vehtari A, Gelman A, Gabry J. 2017. Practical Bayesian model evaluation using leave-one-out cross-validation and WAIC. Stat Comput 27:1413–1432.
